# Activation of Mutant Enzyme Function *In Vivo* by Proteasome Inhibitors and Treatments that Induce Hsp70

**DOI:** 10.1371/journal.pgen.1000807

**Published:** 2010-01-08

**Authors:** Laishram R. Singh, Sapna Gupta, Nicholaas H. Honig, Jan P. Kraus, Warren D. Kruger

**Affiliations:** 1 Cancer Genetics and Signaling Program, Fox Chase Cancer Center, Philadelphia, Pennsylvania, United States of America; 2 Department of Pediatrics, University of Colorado School of Medicine, Aurora, Colorado, United States of America; Stanford University School of Medicine, United States of America

## Abstract

Missense mutant proteins, such as those produced in individuals with genetic diseases, are often misfolded and subject to processing by intracellular quality control systems. Previously, we have shown using a yeast system that enzymatic function could be restored to I278T cystathionine β-synthase (CBS), a cause of homocystinuria, by treatments that affect the intracellular chaperone environment. Here, we extend these studies and show that it is possible to restore significant levels of enzyme activity to 17 of 18 (94%) disease causing missense mutations in human cystathionine β-synthase (CBS) expressed in *Saccharomyces cerevisiae* by exposure to ethanol, proteasome inhibitors, or deletion of the Hsp26 small heat shock protein. All three of these treatments induce Hsp70, which is necessary but not sufficient for rescue. In addition to CBS, these same treatments can rescue disease-causing mutations in human p53 and the methylene tetrahydrofolate reductase gene. These findings do not appear restricted to *S. cerevisiae*, as proteasome inhibitors can restore significant CBS enzymatic activity to CBS alleles expressed in fibroblasts derived from homocystinuric patients and in a mouse model for homocystinuria that expresses human I278T CBS. These findings suggest that proteasome inhibitors and other Hsp70 inducing agents may be useful in the treatment of a variety of genetic diseases caused by missense mutations.

## Author Summary

Genetic diseases are often caused by missense mutations: single nucleotide changes that cause a single incorrect amino acid to be substituted into the underlying protein. Most missense mutations cause the encoded protein to fold incorrectly and therefore not function properly. Examples of missense mutation diseases include CBS deficiency, Li-Fraumeni syndrome, and methylenetetrahydrofolate reductase deficiency, which are caused by alterations in the *CBS*, *TP53*, and *MTHFR* genes, respectively. In the work presented here, we show that it is possible to restore substantial enzymatic function to disease-causing human mutant CBS, p53, and MTHFR proteins by treatments that alter the intracellular folding environment. All of these treatments result in elevation of the Hsp70 protein, a molecular chaperone protein involved in helping proteins fold properly. One way to rescue mutant protein function is by using a drug that inhibits the function of the proteasome, the intracellular machine responsible for degrading misfolded proteins. Our findings suggest that drugs of this class may be potentially useful in the treatment of human genetic diseases caused by missense mutations.

## Introduction

Missense mutations are genetic alterations that result in the production of proteins with single amino acid changes and are an especially common cause of a variety of diseases [1]. Most disease causing missense mutations do not target key catalytic residues, but rather cause problems in protein folding. It is thought that missense mutations affect protein folding by “trapping” the protein in a non-functional intermediate state, preventing it from folding into its lowest-free energy native state. These trapped misfolded protein intermediates can either be degraded or form large molecular weight aggregates [2]. In theory, treatments that could reverse these protein-folding defects and promote proper folding would be of great utility in the treatment of a wide variety of genetic diseases.

Three genetic diseases in which missense mutations are common include cystathionine β-synthase (CBS) deficiency, Li Fraumeni syndrome, and methylenetetrahydrofolate reductase deficiency. CBS deficiency is an inborn error of sulfur metabolism characterized by very high levels of plasma total homocysteine (tHcy). CBS catalyzes the condensation of homocysteine with serine to form cystathionine and is the first step in the *de novo* production of cysteine. In healthy adults, tHcy concentration in plasma ranges from 5 to 15 µM, but untreated patients with CBS deficiency often have tHcy in excess of 200 µM [3]. CBS deficient patients suffer from various pathologies including arteriosclerosis, osteoporosis, mental retardation, and dislocated lenses [4]. The major cause of mortality in these patients is stroke. Treatments that lower tHcy such as B-vitamins, dietary methionine restriction, and betaine supplementation, can significantly reduce the incidences of vascular events in these patients despite the fact that post-treatment homocysteine levels are still several times higher than levels found in the normal population [5],[6],[7]. Mouse models for CBS deficiency also indicate that there is a threshold effect for tHcy toxicity and support the notion that a small increase in residual CBS activity may have large clinical benefits [8].

Li-Fraumeni syndrome is a dominant cancer susceptibility syndrome disorder caused by missense mutations in the *TP53* tumor suppressor gene [9]. Li-Fraumeni patients suffer from a variety of cancers, including sarcomas, adrenocorticol carcinomas, breast cancer, leukemia, and brain tumors [10]. In general, *TP53* behaves as a classic tumor suppressor gene, with the tumors losing or inactivating the wild-type copy of *TP53*, resulting in expression of only the mutant form. Mutant forms of p53 tend to be stable, resulting in increased accumulation of protein [11]. Of the 165 mutations in the *TP53* gene described in the Human Gene Mutation Database [12], 110 are of the missense variety (68%).

MTHFR is a critical enzyme in the remethylation of homocysteine to methionine. Its biochemical function is to catalyze the formation of 5-methyltetrahydrofolate, which is the methyl-group donor for the subsequent reaction catalyzed by methionine synthase. Mutations in *MTHFR* are known to cause MTHFR deficiency. MTHFR deficiency symptoms include developmental delay, motor or gait abnormalities, seizures, and premature vascular disease [13]. Thirty-four mutations have been described in MTHFR deficient patients, and 23 are predicted to encode missense mutations (67%) [12].

Previously, work from our lab has shown that it is possible to restore significant enzymatic function to human CBS containing an isoleucine to threonine substitution at position 278 (I278T) by growth of cells in ethanol containing media [14]. This rescue was shown to require the induction of Hsp70, a key cellular chaperone protein [15]. Hsp70 protein interacts with misfolded polypeptides along with co-chaperones and promotes refolding by repeated cycles of binding and release requiring the hydrolysis of ATP [16]. In the work described here, we have extended our observations by showing that treatments that induce Hsp70 can greatly increase enzymatic function from 17 additional mutant CBS proteins, as well as mutant forms of p53 and MTHFR. In addition we show that proteasome inhibitors can induce Hsp70 and that these drugs can greatly increase mutant CBS activity in human cells and in a mouse model of CBS deficiency. Our findings support the idea that drugs that induce Hsp70 may be useful in the treatment of genetic disorders caused by missense mutations.

## Results

### Ethanol and deletion of *HSP26* restores enzymatic activity to mutant human CBS

**Figure 1:**
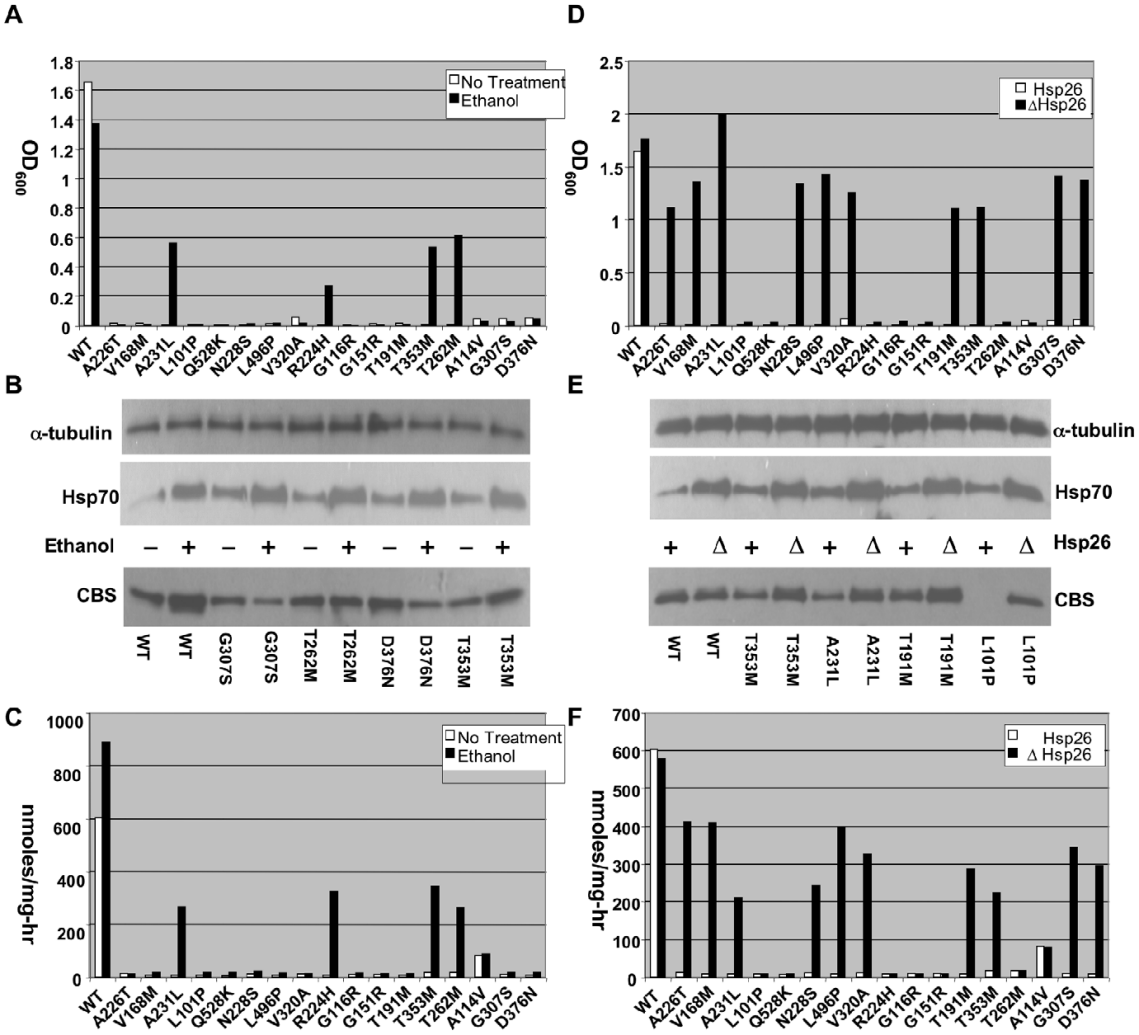
Functional rescue of mutant CBS by ethanol and *hsp26Δ*. (A) A stationary phase culture of yeast (WY35) transformed with a plasmid expressing the indicated human CBS allele was diluted 1∶1000 in SC–Cys media with or without 4% ethanol and grown at 30°C for 24 h. Growth was determined by measuring OD at 600 nm. (B). Western analysis of two representative ethanol rescuable (T353M, T262M) and two non-rescuable (G307S, D376N) mutants. Cells were grown in SC+CYS media with or without ethanol and extracts were probed with indicated antibody. (C) CBS enzyme activity of each of the mutant alleles in the presence and absence of ethanol. (D) Growth of *HSP26* (WY35) or *hsp26Δ* (LS2) yeast expressing the indicated CBS allele. (E) Western analysis of two representative *hsp26Δ* rescuable (T353M, A231L) and two non-rescuable (T191M, L101P) mutants. (F) CBS enzyme activity of each of the mutant alleles in the presence and absence of *Hsp26*.

We initially examined the effect of ethanol on a panel of 17 additional missense *CBS* mutations found in homocystinuric patients [17]. Each of these mutations was expressed in a yeast strain (WY35) that is deleted for the endogenous *CBS* gene (*cys4Δ*) and growth was examined on cysteine-free media either lacking or containing 4% ethanol (Figure 1A). In addition, we prepared total cellular lysates from the strains grown in cysteine-supplemented media with or without ethanol and measured both the steady state level of each mutant protein by Western blot and CBS enzyme activity (Figure 1B and 1C, 1, Table 1). We found that 4/17 (24%) of the mutants exhibited significant growth and greatly increased CBS enzyme activity (8 to 50-fold) when grown in ethanol-containing media. Interestingly, like I278T, ethanol had a much more modest effect on steady state protein levels compared to enzyme activity, indicating that the treatment caused the specific activity of the enzyme to increase. Ethanol exposure also increased steady state Hsp70 levels, consistent with our previous observations (Figure 1B).

**Table 1:** Relative levels are shown for CBS protein and activity in yeast.

Protein Levels					Activity Levels			
Mutants	No treatment	Ethanol	Bortezomib	ΔHsp26	No treatment	Ethanol	Bortezomib	ΔHsp26
WT	1.0	2.2	1.6	1.2	100	149	120	96
G307S	0.9	0.4	2.4	1.1	1	3	2	57
T262M	1.1	1.2	2.2	1.2	3	44	68	3
D376N	1.4	0.6	2.2	1.4	1	3	2	50
T353M	0.7	1.1	2.3	1.6	3	25	44	37
A231L	0.3	1.4	2.1	1.2	1	45	3	35
T191M	0.7	0.3	6.1	1.7	1	4	2	48
G151R	0.7	0.2	1.6	0.8	1	4	35	1
L101P	0	0	1.2	0.9	1	4	28	1
N228S	1.4	0.7	2.1	1.6	2	4	29	40
Q528K	1.5	0.7	2.8	2.3	1	3	17	1
L496P	0	0	1.9	0.9	1	3	2	66
G116R	0	0	1.2	0	1	3	3	1
A114V	0	0	ND	ND	14	15	70	13
V320A	1.5	0.8	1.8	1.8	2	2	84	54
R224H	0.3	0.3	2.2	1.2	1	55	28	1
V168M	0.3	0.3	1.9	1.0	1	4	2	67
A226T	1.0	ND	1.6	1.6	2	2	22	68
I278T	0.2	0.6	1.4	0.6	2	65	49	59

**Protein is normalized to 1 while enzyme activity is normalized to 100 for wild-type human CBS.**

Previously, we had also shown that deletion of the small heat shock protein *HSP26* (*hsp26Δ*) could effectively suppress the functional effects of I278T CBS mutation in yeast [14]. Therefore, we introduced our panel of additional missense mutants into a *cys4Δ hsp26Δ* double mutant strain and examined function. As with ethanol, *hsp26Δ* resulted in increased steady state levels of Hsp70 (Figure 1E). Deletion of *Hsp26* could rescue the cysteine growth auxotrophy and restore significant levels of enzymatic activity to 10/17 (59%) of the mutants tested (Figure 1D–1F, Figure S2, Table 1). Again, the increased level of enzyme activity in the suppressible mutants was impressive, ranging from an 8 to 55-fold increase resulting in levels that were between 25% and 58% of wild-type CBS. Like ethanol, most of the increased enzyme activity appears to be due to increased functionality of the mutant protein as opposed in increased protein levels.

In our earlier work, we had shown that expression of I278T CBS drove down levels of Hsp26 protein and that I278T CBS, but not wild-type CBS, physically interacted with Hsp26 [14]. We tested two of the newer mutants (G307S, D376N) for these properties and found that they also caused decreased steady state levels of Hsp26 and co-immunoprecipitated Hsp26 (Figure S3). These results indicate that like I278T, G307S and D376N form a complex with Hsp26.

### Bortezomib rescues several forms of mutant human CBS expressed in *S. cerevisiae*

**Figure 2:**
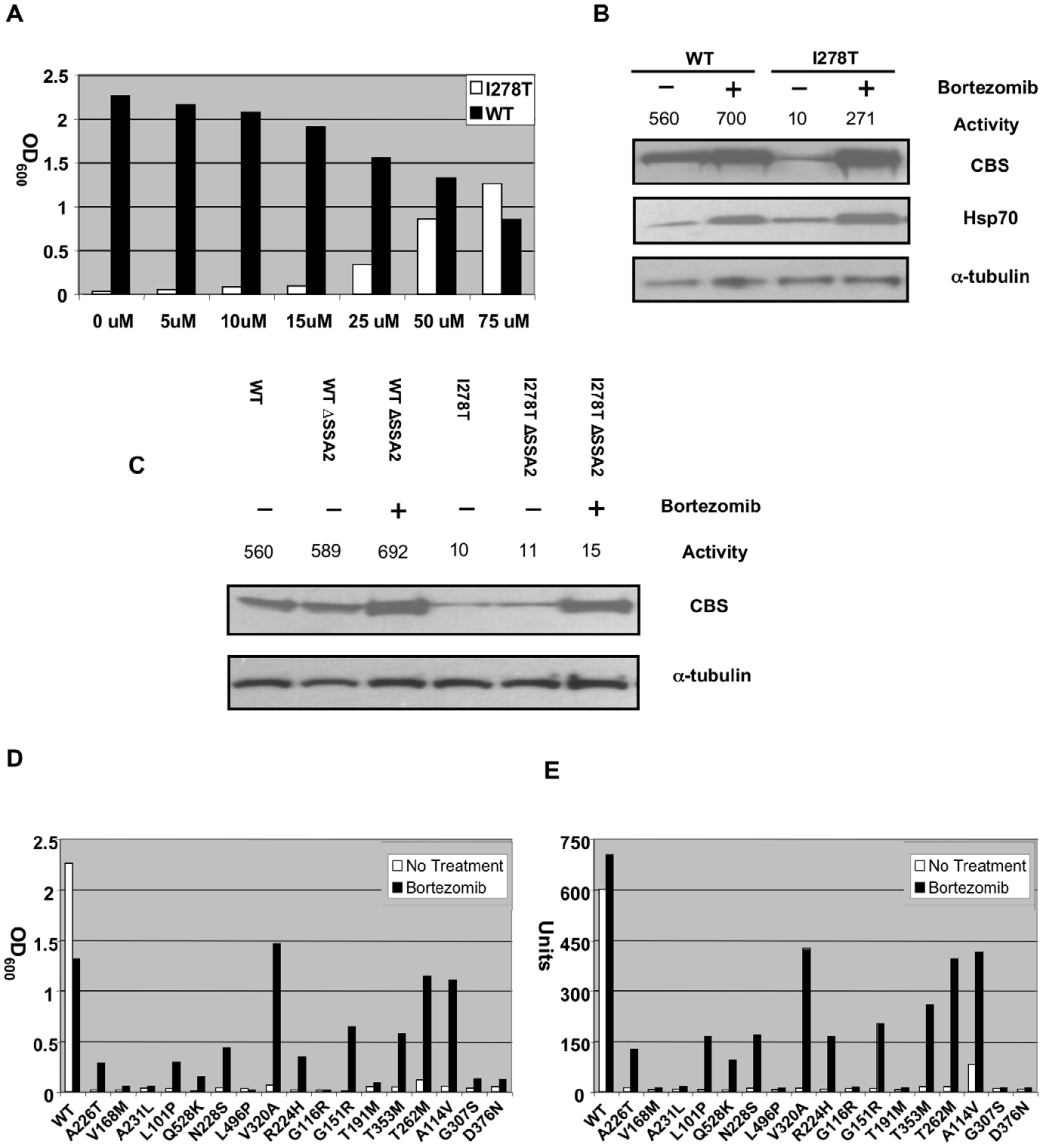
Functional rescue of mutant human CBS expressed in *S. cerevisiae* by bortezomib treatment. (A) A stationary phase culture of yeast (WY35) transformed with a plasmid expressing either WT (phCBS) or I278T (pI278T) human CBS was diluted 1∶1000 in SC–Cys media with the indicated amount of bortezomib and grown at 30°C for 24 h. Growth was determined by measuring OD at 600 nm. (B) WY35 pI278T and WY35 phCBS cells were grown in SC–Trp+Cys media either with or without 50 µM of bortezomib for 24 hours and extracts were prepared. Levels of CBS, α-tubulin, and Hsp70 and CBS activity were assessed as described in Materials and Methods. CBS units are nmole cystathionine formed/mg protein-hr. (C) Yeast strain WY35 phCBS, WY35 pI278T, LS3 phCBS, LS3 pI278T were grown at 30°C in the absence and presence of bortezomib and extracts were assessed for CBS activity and CBS protein. (D) Stationary phase cultures of yeast strain WY35 expressing the indicated allele of CBS were diluted 1∶1000 in SC–Cys media either with or without 50 µM of bortezomib and growth was monitored by OD_600_ after 24 h at 30°C. (E) Log phase cultures of WY35 grown in SC+Cys media with or without 50 µM of bortezomib expressing the indicated CBS allele were harvested, lystates prepared, and CBS enzyme activity was measured and expressed in units.

Since proteasome inhibitors have previously been shown to induce Hsp70 in both yeast and mammalian cells [18],[19], we tested if these agents might be able to restore function to missense mutant CBS proteins *in vivo*. Bortezomib (also known as PS-341 or Velcade) is a potent proteasome inhibitor that is currently used to treat humans with multiple myeloma [20]. We first tested the effects of bortezomib on mutant human I278T CBS function in yeast. We found that addition of bortezomib to yeast media strongly rescued, in a dose-dependent fashion, the cysteine auxotrophy of WY35 cells carrying a plasmid expressing human I278T CBS (pI278T) (Figure 2A). After 24 hours, 75 µM bortezomib allowed WY35pI278T cells to achieve growth that was at least equivalent to WY35 expressing wild type human CBS (phCBS). Examination of I278T protein and activity from treated vs. untreated cells indicated that bortezomib increased steady-state levels of I278T by 7-fold and increased I278T activity by 27-fold, to about 50% of that observed in untreated cells expressing wild-type CBS (Figure 2B). These results show that bortezomib can both stabilize and restore function to I278T CBS expressed in yeast.

Bortezomib treatment also caused a 2.8 to 3.6-fold increase in Hsp70 in WT and I278T expressing strains, respectively (Figure 2B). To determine if this induction was essential for restoration of I278T CBS function, we examined the effect of bortezomib on yeast lacking the Hsp70-encoding gene *SSA2* (*ssa2Δ*; Figure 2C). As expected, steady state levels and the enzyme activity of I278T CBS were lower compared to wt CBS in both *SSA2* and *ssa2Δ* cells in the absence of bortezomib. In the presence of bortezomib, we saw stabilization of I278T protein in *ssa2Δ* cells, but no increase in enzyme activity. In *ssa2Δ* cells expressing WT CBS, we did not observe any decrease in enzyme activity, indicating that *SSA2* is not required generally for human CBS function. These results show that simply blocking proteolysis of I278T CBS is not sufficient to restore function to the mutant enzyme, and that *SSA2* (Hsp70 protein) is required for bortezomib-induced rescue of function.

We also investigated the effect of bortezomib on our panel of other patient-derived CBS mutants. We found that 9/17 of the alleles tested showed significant growth in cysteine-free media and had enzyme activity restored to between 37–68% of wild-type CBS (Figure 2D). It should be noted that some of these mutants (T353M and T262M for example) produce stable but non-functional enzymes, indicating that bortezomib is not simply working by preventing protein degradation (Figure S4)

### Restoration of function to mutant forms of p53 and MTHFR

**Figure 3:**
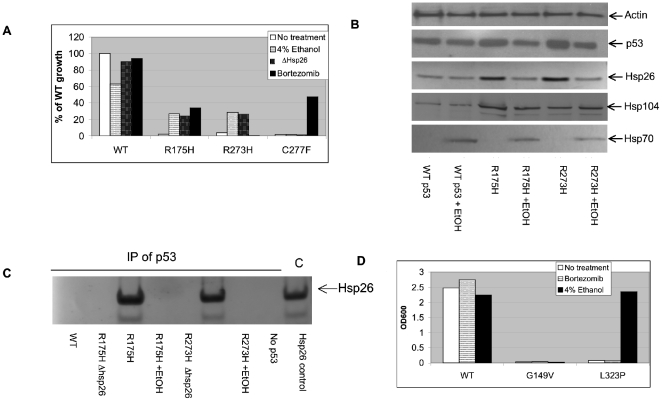
Functional rescue of mutant p53 and MTHFR in yeast. (A) Yeast p53 tester strain yIG397 (*HSP26*), or LS2 (hsp26Δ) were transformed with an expression plasmid expressing the indicated p53 alleles. Stationary phase cultures of yeast expressing the wild type and indicated mutant were diluted 1∶1000 in SC-ura -ade media grown in 15 ml tubes with aeration at 30°C for 24 hours. Where indicated, 4% ethanol or 50 µM bortezomib were added to the media. (B) Yeast strain yIG397 carrying the indicated p53 alleles was grown in adenine containing media with or without 4% ethanol. Lysates were then probed by Western Blot with the indicated anti-bodies. (C) Yeast strains yIG397 and LS2 expressing the indicated p53 alleles were grown in adenine containing media supplemented with 4% ethanol when indicated. Lysates were prepared and immunoprecipitation was performed with anti-p53 antibody and Hsp26 present in the immune complexes was analyzed by immunoblot. (D) Yeast MTHFR tester strain XSY3-1A (*met11Δ*) were transformed with expression plasmids expressing the indicated MTHFR allele.

We next determined if chaperone manipulation could be used to restore function to disease causing mutants of p53 and MTHFR. To examine p53 function, we used a yeast strain (yIG397) in which human p53 binds upstream of the *ADE2* promoter, activates transcription, and results in correction of the strains adenine auxotrophy [21]. Into this strain we transfected plasmids that expressed either wild-type human p53 or one of three different patient-identified point mutations (R175H, R273H, C277F) and measured p53 function by examining growth in adenine deficient media. As expected, we found that cells expressing wild type human p53 grew well, while cells expressing the mutant alleles had almost undetectable levels of growth (Figure 3A). However, when the media was supplemented with either 4% ethanol, 50 µM Bortezomib, or an *hsp26Δ* construct was introduced into the strain, each mutant exhibited significant growth rescue with at least one of the treatments.

We performed more in depth analysis on two of the mutant alleles, R175H and R273H. In the absence of ethanol, steady state levels of both of these mutant proteins are slightly elevated compared to wild type CBS and cause elevations in the steady state levels of Hsp26 and Hsp104 (Figure 3B). The elevation in Hsp104 was unexpected, as we had previously shown that I278T CBS had no effect on Hsp104 levels [14]. When ethanol is added to the cultures, Hsp70 levels are induced and p53 and Hsp26 levels are reduced to wild-type levels, while hsp104 levels remain elevated. The observation that p53 levels go down, while growth increases indicates that, like CBS, ethanol is enhancing the specific activity of p53.

We also examined the dependence of ethanol rescue on having a functionally intact *SSA2* and *HSP104* (Figure S5). Deletion of either gene resulted in total loss of the ability of ethanol to promote adenine independent growth. Immunoprecipitation experiments demonstrate that, similar to mutant CBS, Hsp26 recognizes mutant p53 but not wild-type p53 and that this interaction is lost upon ethanol treatment. (Figure 3C). Taken together, these results show that p53 mutants behave similar, but not identical, to CBS mutants with regards to rescue by agents that perturb the cellular chaperone environment.

We next determined the frequency by which defective p53 protein could be rescued by either ethanol or bortezomib treatment. We examined rescue using a panel of 22 single missense mutant p53 alleles that were obtained by random mutagenesis of the human p53 (see Materials and Methods). Like CBS, we found that some mutants were rescuable by both ethanol and bortezomib (4/22), some only by bortezomib (8/22), some only by ethanol (4/22), and some that were not rescuable by either treatment (6/22). In total, we found that 16 out of 22 (72%) of the randomly generated mutants were rescuable by at least one of the treatments (Figure S6).

We have also examined the effect of ethanol and bortezomib on mutant alleles of human 5–10-methylene tetrahydrofolate reductase (MTHFR) expressed in yeast. In this assay, expression of a functional human MTHFR enzyme complements the methionine auxotrophy present in a *met11Δ* mutant [22]. Two missense mutant MTHFR proteins were tested for growth rescue by either addition of ethanol or bortezomib and we found that one, L323P, could be rescued by the addition of ethanol (Figure 3D).

### Restoration of CBS function in patient fibroblasts by MG132

**Figure 4:**
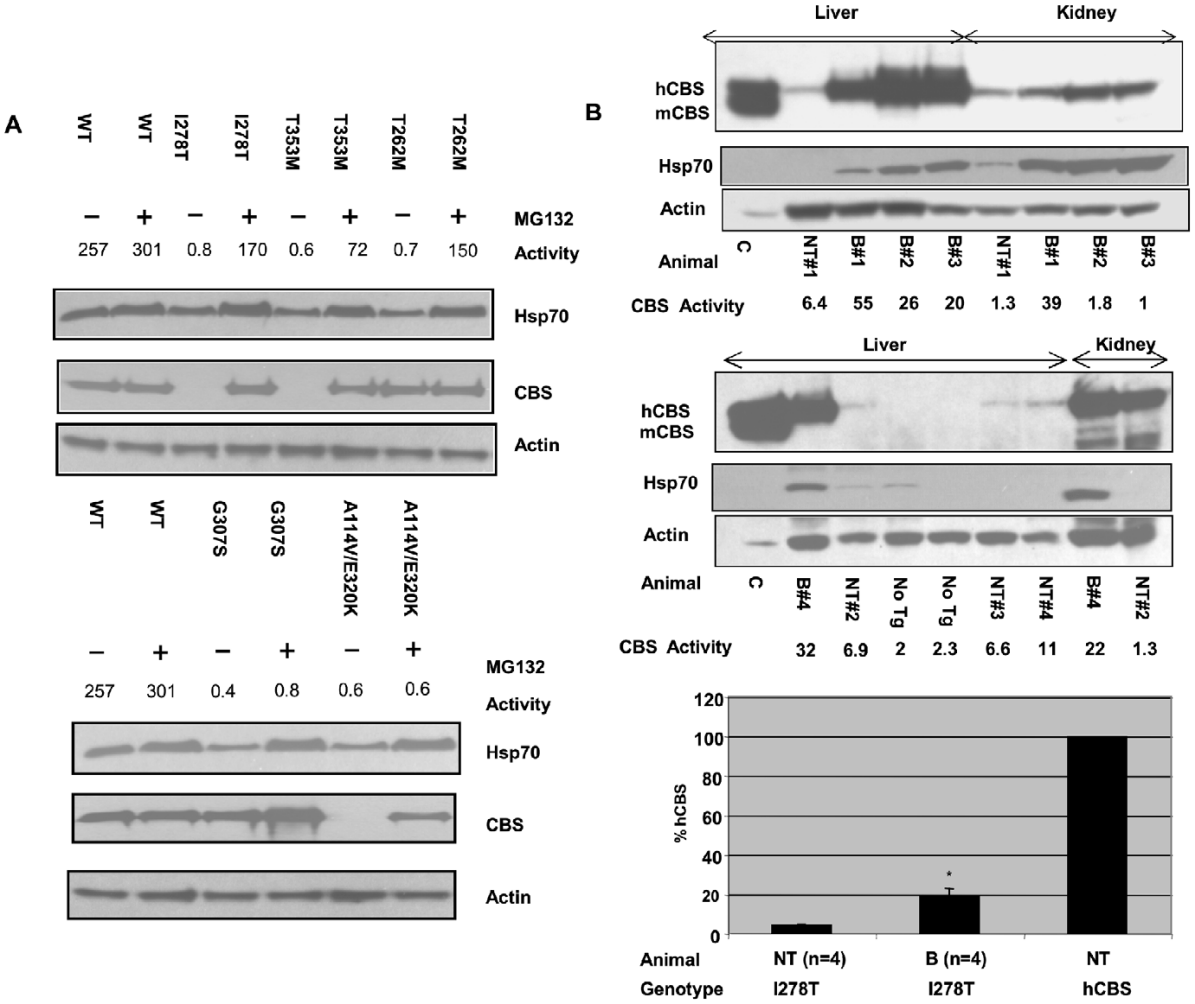
Functional rescue of mutant CBS by proteosome inhibitors in mammalian cells. (A) Restoration of CBS enzyme activity in patient fibroblasts. Primary fibroblast cells expressing the indicated CBS allele were grown to 80% confluency at which time MG132 (18 µM) was added. After seven hours protein was harvested and Hsp70, CBS, and actin protein levels were measured by immunoblot. CBS activity was measured and activity is expressed as nmole cystathionine formed/mg protein-hr. Note that the G307S cell line is a transformed lymphoid cell line. (B) Effect of intravenous bortezomib treatment on *Tg-I278T Cbs^−/−^* mice. *Tg-I278T Cbs^−/−^* mice were kept on zinc water for 10 days to induce transgene expression and then either injected with bortezomib (30 µg) or saline by tail vein injection. After 17 hours mice were sacrificed and tissues harvested. Immunoblot analysis of CBS in liver and kidney extracts from bortezomib treated “B” and non-treated (NT) I278T mice. Actin was used as loading control. CBS activity was measured and activity is expressed as nmole cystathionine formed/mg protein-hr. Mice labeled “No Tg” are *Cbs^−/^* ^−^ animals lacking human CBS. Lane labeled “C” contains extract from a *Tg-I278T Cbs^+/−^* animal. Bar graph shows Liver CBS activity in bortezomib treated (n = 4) and non-treated (n = 4) *Tg-I278T Cbs^−/−^*mice was compared with CBS activity in a non-treated mouse containing normal hCBS. Statistical significance was calculated by t test. Results are represented in terms of mean±SE. *P<0.05.

We next determined if the restoration of function we observed in *S. cerevisiae* could also occur in mammalian cells. To test this, we examined the effect of proteasome inhibitors on mammalian cells expressing mutant human CBS protein. We obtained four primary fibroblast lines and one EBV transformed lymphoblastoid line from five patients with CBS deficiency. Four of the five lines were from patients that were homozygous for a particular mutation (I278T, T353M, T262M, G307S), while one line was from a compound heterozygote (A114V/E302K). We then examined both steady state CBS protein and CBS enzyme activity in untreated and in cells treated with the proteasome inhibitor MG132 (Figure 4A). MG132 was used in these experiments because it was found to be a more potent inducer of Hsp70 than bortezomib for these cells (Figure S7). In the absence of drug, three of the five lines (I278T, T253M, and A114V/E302K) had undetectable levels of CBS protein and all five lines had <1% enzyme activity compared to a control primary human fibroblast line. Addition of MG132 resulted in restoration of steady-state CBS to wild-type levels in all five lines and caused a dramatic increase in enzyme activity (over 100-fold increase to 28 to 66% of wt CBS) in three of the five lines (I278T, T353M, and T262M). Interestingly, the increase in enzyme activity in the T262M cells was not associated with increased T262M protein levels, again suggesting that the rescuing effect of proteasome inhibitors is not due to inhibition of mutant protein degradation. Consistent with this idea, we found that MG132 resulted in a two-fold increase in Hsp70 (Figure 4A). With one exception, we note concordance in the behavior of mutants that were rescuable by proteasome inhibition in yeast and in patient fibroblasts. It is possible that the lack of rescue observed in the A114V allele may have to do with the cell line also having the E320K allele as well. In total, these results indicate that exposure of human cells to proteasome inhibitors can rescue enzymatic activity from several missense mutant CBS proteins.

### Increased CBS activity and lower plasma homocysteine in *Tg-I278T Cbs^−/−^* mouse treated with bortezomib

Previously we had developed a mouse model for homocystinuria caused by I278T CBS (*Tg-I278T Cbs^−/−^*). This mouse contains a homozygous deletion of the mouse *CBS* gene, and expresses human I278T CBS under control of a zinc-inducible promoter [23]. In this animal it was demonstrated that human I278T CBS expressed in mouse liver had minimal enzyme activity [24]. To test the effect of bortezomib *in vivo*, we induced I278T expression by addition of zinc to the drinking water, and after one week injected 30 µg of bortezomib into the tail veins of four *Tg-I278T Cbs^−/−^* animals. After 17 hours, serum, liver and kidney were then harvested and analyzed. Examination of steady state CBS protein indicates that bortezomib greatly increased the amount of liver I278T protein and Hsp70 protein compared to a mock-injected control (Figure 4B). In addition, CBS enzyme activity increased on average 4.3-fold in the livers of treated animals compared to untreated controls, to about 18.7% of that found in an animal that expresses wild-type human CBS (Figure 4B). We also observed increased enzyme activity in kidney extracts in two of the four injected animals (Figure 4B). Interestingly, the kidney does not show significantly increased steady-state I278T protein, indicating that the increased enzyme activity is due entirely to increased enzyme specific activity. Bortezomib treatment resulted in a 46% decrease in tHcy in serum, (p<0.05) (Figure S8). Bortezomib also resulted in a 28% decrease in serum methionine, although this was not statistically significant (Figure S8). These results indicate that bortezomib can decrease tHcy *in vivo* by increasing residual I278T enzyme activity.

## Discussion

In the work described here, we have shown that it is possible to restore substantial amount of functional activity to a large percentage of missense mutant alleles of human CBS, p53, and MTHFR expressed in *S. cerevisiae* by treatments that cause Hsp70 induction. These treatments include addition of ethanol or proteosome inhibitor to the media, or deletion of the small heat shock protein *HSP26*. At least one of these treatments was able to dramatically increase enzyme or transcriptional activity in 17/18 (94%) mutant CBS proteins, 19/25 mutant p53 proteins (76%), and 1/2 mutant MTHFR proteins (50%). Interestingly, different types of response to the different rescuing treatments were observed. Some mutants were rescued by all three treatments (e.g. I278T CBS), some by two of three treatments (e.g. T262M CBS), while others only responded to a single treatment (e.g. G307S CBS). These differences are probably not due to differential Hsp70 induction, as all three treatments result in similar levels of induction. We speculate that each of these different treatments may have unique effects on other molecular chaperones and that each treatment may produce a unique intracellular folding milieu. With regards to CBS mutants, we found that both pyridoxine responsive mutants (e.g. I278T) and non-responsive mutants (e.g. G307S) could be rescued.

Our findings support the view that most missense mutations primarily affect protein folding, as opposed to altering critical residues involved in specific catalytic or interaction sites. This agrees with biophysical studies of protein folding. As stated by DePristo et al. [25] “The overwhelming conclusion from 20 years of mutation studies on protein stability is that most amino-acid replacements, at all sites in a protein result in large effects on ΔG relative to the observed range of ΔG values themselves.” It is also possible that in multi-domain proteins like CBS, mutations affect protein folding by trapping the protein in a non functional intermediate state due to the existence of a high kinetic barrier [2]. Therefore, a possible explanation for the effectiveness of chaperone manipulation is that chaperone complex either helps mutant proteins overcome rate-limiting kinetic constraints in the folding process or brings the trapped intermediate back to a folding competent state. Interestingly, in CBS, only one mutation, G116R, was not rescuable by any treatment. This mutation is located only three residues away from the lysine (K119) that binds the active site pyridoxal phosphate at the base of an alpha helix [26]. Thus this change may be an example of a mutation that affects a key catalytic-site in the protein.

Our results also show that this is not a yeast-specific phenomenon. We were able to restore function to three of six mutant alleles present in fibroblast cells from CBS deficient patients by treatment with the proteasome inhibitor MG-132. In addition, we showed that a 4.3-fold increase in CBS activity could be achieved with a single injection of bortezomib into mice expressing I278T CBS. Consistent with our findings, Mu *et al.* showed that MG-132 could increase residual glucocerebrosidase enzyme activity four-fold in cell lines containing the L444P variant associated with Gaucher disease, and that a combination of MG-132 and a chemical chaperone, 2-Acetamido-2-deoxynojirimycin, could increase residual activity five-fold in cells containing the Tay-Sachs disease causing α-hexosaminidase G269S mutation [27]. In addition, the Hsp70-inducing drug arimoclomol was reported to delay disease progression in mice expressing a SOD1 mutant in which glycine is substituted with alanine at position 93 [28].

Taken together, these findings suggest that treatment with proteasome inhibitors and other Hsp70 inducers may be beneficial to individuals with severe genetic diseases caused by missense mutation. One potential concern with drugs that manipulate the molecular chaperone environment is they may have potential adverse effects on the normal proteome, and thus create side effects that would preclude their use as drugs. While this is a possibility, it should be noted that Bortezomib is already an FDA approved drug that is used to treat multiple myeloma. The major noted toxicity is peripheral neuropathy [29]. However, it is unknown if the levels of Bortezomib used to induce changes in the chaperone environment would need to be as high as those used in chemotherapy. Furthermore, there is another proteasome inhibitor in clinical trials (PR-171) that does not exhibit a strong peripheral neuropathy effect [30]. In addition, there are at least two other drugs that are known Hsp70 inducers that have undergone successful phase I trials in humans including 17-allylamino-17-demethoxygeldanamycin and arimoclomol [31],[32]. Therefore, while there may be some risk in this approach, there is no reason *a priori* to believe that the benefits of restoring function to a mutant protein would not out weigh the potential side effects.

In addition to germline genetic diseases, the work described here may have relevance to the treatment of somatic genetic diseases such as cancer. Mutations in p53 are the most frequent genetic alteration in human cancer, and loss of p53 function is critical for tumorigenesis [33]. Several pharmacologic chaperones that restore function to mutant p53 protein have been identified and these have been shown to effectively induce apoptosis in certain cells that express mutant p53 [34],[35]. Our findings suggest that proteasome inhibitors may also effectively restore function to certain p53 alleles. Although bortezomib has been shown to inhibit cell growth and cause apoptosis in a large number of cancer cell lines, the IC_50_ concentrations vary widely [36]. No systematic study of the relationship between bortezomib sensitivity and the p53 status of tumor cells has been reported.

In summary, the data reported here shows that the functional effects of a majority of missense mutations in at least three human genes can be reversed by treatments that alter the intracellular molecular chaperone environment. Based on these results, we suggest that drugs that effect the intracellular chaperone environment may be useful in the treatment of a number of genetic diseases caused by missense mutations.

## Materials and Methods

### Yeast strains, plasmids, growth, and drug treatment

Yeast strains WY35 (α *leu2 ura3 ade2 trp1 cys4*::*LEU2*), LS1 (*α leu2 ura3 ade2 trp1 cys4::LEU2 hsp26::KanMX*) and LS3 (α *leu2 ura3 ade2 trp1 cys4*::*LEU2 ssa2*::*KanMX*) were generated as previously described [14],[37]. Yeast strain yIG397 (*MATa ade2-1 leu2-3,112 trp1-1 his-11,15 can1-100*, *ura3-1 URA3 3XRGC::pCYC1::ADE2*) was obtained from Dr. Richard Iggo [21]. Strains LS3 (yIG397+*hsp26Δ*), LS4 (yIG397+*ssa2Δ*) and LS5 (yIG397+*hsp104Δ*) were derived from this strain by transformation with appropriate deletion cassettes as previously described [14]. The MTHFR tester strain, XSY3-1a (*MATa ade2-1*, *can1-100*, *ura3 leu2 trp1*, *his3*, *trp1 met11Δ::TRP1*) was created as previously described [22]. Plasmids expressing wild-type or mutant human CBS proteins were created by site-directed mutagenesis and gap-repair as described [38],[39]. The plasmids expressing mutant p53 alleles were constructed using gap repair with pRDI-22 [21]. Additional p53 mutants were generated by amplification with *TaqI* polymerase, followed by gap-repair and subsequent screening on SC-ade media for non-functional alleles. Ninety-six clones were then isolated and sequenced. Twenty-three of these clones contained a single missense mutation and were used for the studies described here. Bortezomib (Velcade™, Millennium Pharmaceuticals, Inc) was obtained from the Fox Chase Cancer Center outpatient clinic. Synthetic complete media lacking cysteine (SC–Cys) and (SC–Cys+G418) were made as previously described [14]. SC+Cys media were made by adding glutathione to the indicated SC media at a final concentration of 30 µg/ml. All other chemicals were purchased from Sigma. For yeast cell growth assays, saturated cultures were diluted to an OD_600_ of 0.05 and treated with bortezomib in standard 15-ml borosilicate tubes containing 3 ml of media. Cells were then grown at 30°C with rotating aeration. After 24 hours OD_600_ was determined using a Milton Roy Spectronic 601 spectrophotometer (Ivyland, PA).

### Cell lines and drug treatment

Cell lines used in this study were: WS1 (WT) [40], F5889 (I278T/I278T) [41], 382 (T262M/T262M ) [42], 2242 (A114V/E302K) [43], 3161 (T353M/T353M, JPK unpublished), 3079 (G307S/G307S) [44]. Fibroblasts and EBV transformed lymphoid cells were grown in MEM or RPMI medium supplemented with 15% FBS along with appropriate antibiotics, respectively, in 5% CO_2_ at 37°C. MG132 was purchased from Peptide International (product number, IZL-3175v). MG132 or bortezomib was added to cells when they reached 80% confluency and after seven hours of exposure, the MG132 containing media was discarded, cells were rinsed with PBS, and then harvested for protein extraction.

### Extract preparation and immunoblot analysis

Saturated yeast were diluted to an OD_600_ of 0.05 and cells were harvested when OD was between 0.7 and 0.9 (mid-log phase). Yeast extracts were prepared by mechanical lysis as described previously [45]. Total mammalian protein extracts were prepared using the mammalian protein extraction reagent obtained from Pierce, Rockford, IL (product # 78501). Protein concentration was determined by the Coomassie Blue protein assay reagent (Pierce) using bovine serum albumin as a standard. For immunoblot analysis, yeast extracts containing 20 µg or mammalian cell containing 50 µg of total protein were run on precast Tris-acetate gels (Bio-Rad) at 15 mA and transferred to polyvinylidene difluoride membrane as previously described [14]. Immunopurified mouse anti-CBS was purchased from ABNOVA (catalogue # H00000875-A01) and was used at 1/10,000 dilution. Anti-p53 is a mouse monoclonal anti-body obtained by Calbiochem (cat. #OP43) and was used at a 1/1000 dilution. Yeast Hsp70 monoclonal mouse serum was from AbCAM (catalogue # ab5439) and used at a dilution of 1/5000. Human Hsp70 monoclonal antibody (product # SPA-810D) was used at 1/1000 dilution. Α-Tubulin antibody used here is the same as described previously [14]. For rabbit antiserum, a horseradish peroxidase-conjugated anti-rabbit secondary anti-body was used at a 1/30,000 dilution (Jackson ImmunoResearch, West Grove, PA). Signal was detected by chemiluminescence using SuperSignal West Pico chemiluminescent substrate (Amersham Biosciences) and Chemigenius station (Syngene); signal was quantitated by Alpha Innotech software. Immunopreciptiations were performed as previously described [14].

### Mouse studies

All animal studies were reviewed and approved by the Fox Chase Cancer Center Institutional Animal Care and Use Committee. *Tg-hCBS Cbs^−/−^* and *Tg-I278T Cbs^−/−^* mice were generated as previously described [23],[46]. All mice were from C57BL6 strain background and were fed the rodent chow (Teklad 2018SX; Harlan Teklad, Madison, WI, USA) *ad libitum*. Adult mice (9–12 months old) were injected with 30 µl of bortezomib (1 mg/ml) via tail vein injection. After 17 hour, mice were sacrificed and liver, kidney and serum were extracted. Liver and kidney homogenates were prepared as previously described [46]. Western blotting was done under denaturing conditions as previously described [47].

### CBS activity and metabolite measurement

CBS activity was measured in 30 mg of crude dialyzed protein extracts of liver and kidney, using a Biochrom 30 amino acid analyzer (Biochrom, Cambridge, UK) as described previously [47]. One unit of activity is one µM of cystathionine formed per hour per milligram of protein. The same instrument was employed to measure total homocysteine (tHcy) and methionine in serum as described [8].

## Supporting Information

Figure S1 CBS protein levels in all mutants grown in the presence or absence of ethanol. *Cys4Δ* cells (Wy35) were grown in SC+CYS media either in the absence or presence of 4% ethanol. After strains reached an OD_600_∼1.5, extracts were prepared and Western analysis was performed using CBS antibody.
(0.28 MB PDF)

Figure S2 CBS protein levels in all mutants grown in the presence or absence of Hsp26. The indicated mutants were expressed in either a *cys4Δ* strain (Wy35) or a *cys4Δhsp26Δ* strain were grown in SC+CYS media, extracts were prepared, and Western analysis was performed using CBS antibody.
(0.40 MB PDF)

Figure S3 Interaction of D376N and G307S CBS with Hsp26. (A) The indicated mutants were expressed in either a *cys4Δ* strain (Wy35) or a *cys4Δhsp26Δ* (LS1) strain. Cells were grown in SC+CYS media, extracts were prepared, and Western analysis was performed using CBS, Hsp26, and α-tubulin anti-bodies. The CBS enzyme activity present in each extract is shown at the bottom (n = 3; standard deviation shown). (B) Lysates from cells expressing the indicated human CBS allele were prepared and subject to immunoprecipitation using Hsp26 directed anti-body. Immunocomplexes were then analyzed by Western blot with CBS anti-bodies. Lane labeled control is extract without IP.
(0.19 MB PDF)

Figure S4 CBS protein levels in all mutants grown in the presence or absence of bortezomib. The indicated mutants were expressed in a *cys4Δ* strain (Wy35) grown in SC+CYS media in the presence or absence of 50 µM bortezomib. After 24 hours, extracts were prepared and Western analysis was performed using CBS antibody.
(0.70 MB PDF)

Figure S5 Effect of *ssa2Δ* and *hsp104Δ* on ethanol rescue of mutant p53. (A) *SSA2* or *ssa2Δ* yeast cells expressing the indicated p53 alleles were grown in either in the presence or absence of ethanol and assessed for growth in SC-ade-ura media after 24 hours by measuring OD_600_. (B) *HSP104* or *hsp104Δ* yeast cells expressing the indicated p53 alleles were grown in either in the presence or absence of ethanol and assessed for growth in SC-ade-ura media after 24 hours by measuring OD_600_.
(0.16 MB PDF)

Figure S6 Rescue of multiple mutant p53 by ethanol or bortezomib treatment. Stationary phase culture of yeast yIG397 expressing the indicated human p53 allele was diluted 1∶1000 in SC–ade -ura media supplemented with either 4% ethanol or 50 µM bortezomib and grown at 30°C for 24 h. Growth was then determined by measuring OD at 600 nm.
(0.07 MB PDF)

Figure S7 CBS protein and activity in human cells treated with bortezomib. (A) Primary fibroblast cells expressing the indicated CBS allele were grown to 80% confluency at which time Bortezomib (18 µM) was added. After seven hours protein was harvested and Hsp70, CBS, and actin protein levels were measured by immunoblot. (B) Comparison of Hsp70 induction in bortezomib vs. MG132 cells. Gels were scanned using imaging software (Alpha Innotech 2.0) and the average induction is shown. Error bars show standard deviation. P<2.0×10^−5^ (t-test, two-sided).
(0.64 MB PDF)

Figure S8 Serum total homocysteine and methionine levels in Tg-I278T Cbs−/− animals treated with bortezomib. Error bars show standard deviation *P<0.05 (t-test, 2-sided).
(0.05 MB PDF)
