# Supplementary material for: Activation of Mutant Enzyme Function In Vivo by Proteasome Inhibitors and Treatments that Induce Hsp70
Source: PLoS Genet. 2010 Jan 8;6(1):e1000807. doi: 10.1371/journal.pgen.1000807 (PMC2795852; doi:10.1371/journal.pgen.1000807)

SUPPORTING ONLINE MATERIALS

Supplemental Figures

Supp. Fig. 1. CBS protein levels in all mutants grown in the presence or absence of ethanol. *Cys4Δ* cells (Wy35) were grown in SC+CYS media either in the absence or presence of 4% ethanol. After strains reached an OD_{600} ~1.5, extracts were prepared and Western analysis was performed using CBS antibody.

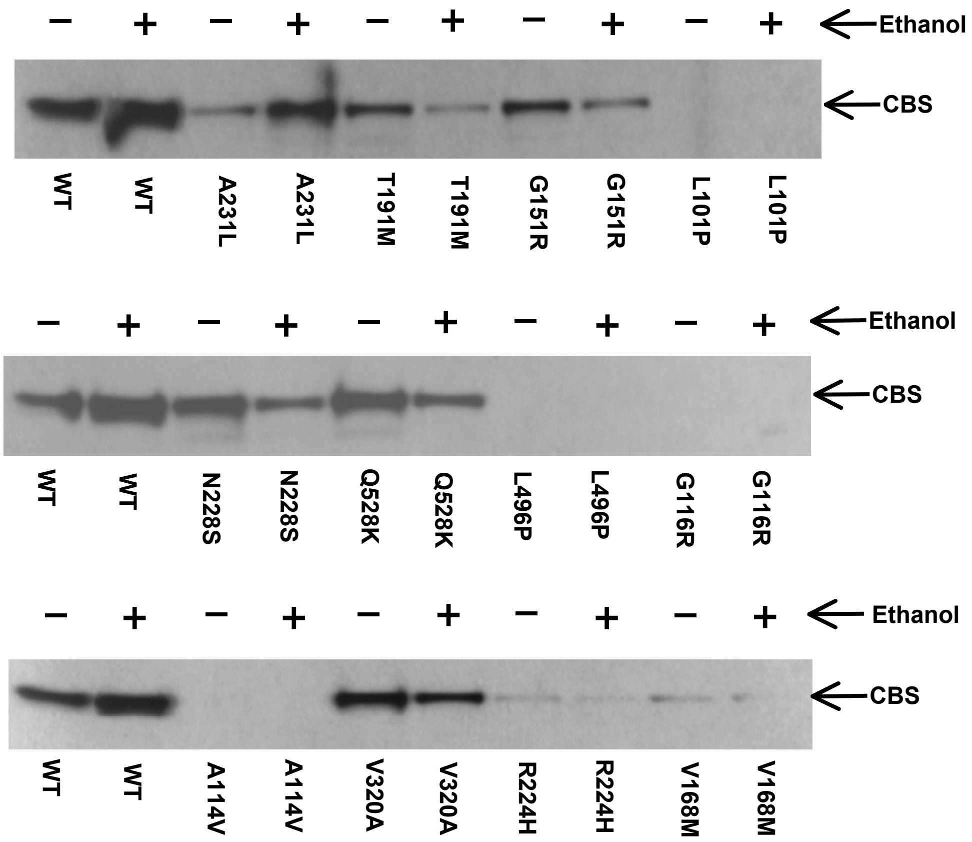

Supplement: Figure S1 — CBS protein levels in all mutants grown in the presence or absence of ethanol. Cys4Δ cells (Wy35) were grown in SC+CYS media either in the absence or presence of 4% ethanol. After strains reached an OD600∼1.5, extracts were prepared and Western analysis was performed using CBS antibody. (0.28 MB PDF) [file pgen.1000807.s001.pdf]
