# Supplementary material for: Activation of Mutant Enzyme Function In Vivo by Proteasome Inhibitors and Treatments that Induce Hsp70
Source: PLoS Genet. 2010 Jan 8;6(1):e1000807. doi: 10.1371/journal.pgen.1000807 (PMC2795852; doi:10.1371/journal.pgen.1000807)

**Supp. Fig. 2**. CBS protein levels in all mutants grown in the presence or absence of Hsp26. The indicated mutants were expressed in either a *cys4Δ* strain (Wy35) or a *cys4Δhsp26Δ* strain were grown in SC+CYS media, extracts were prepared, and Western analysis was performed using CBS antibody.

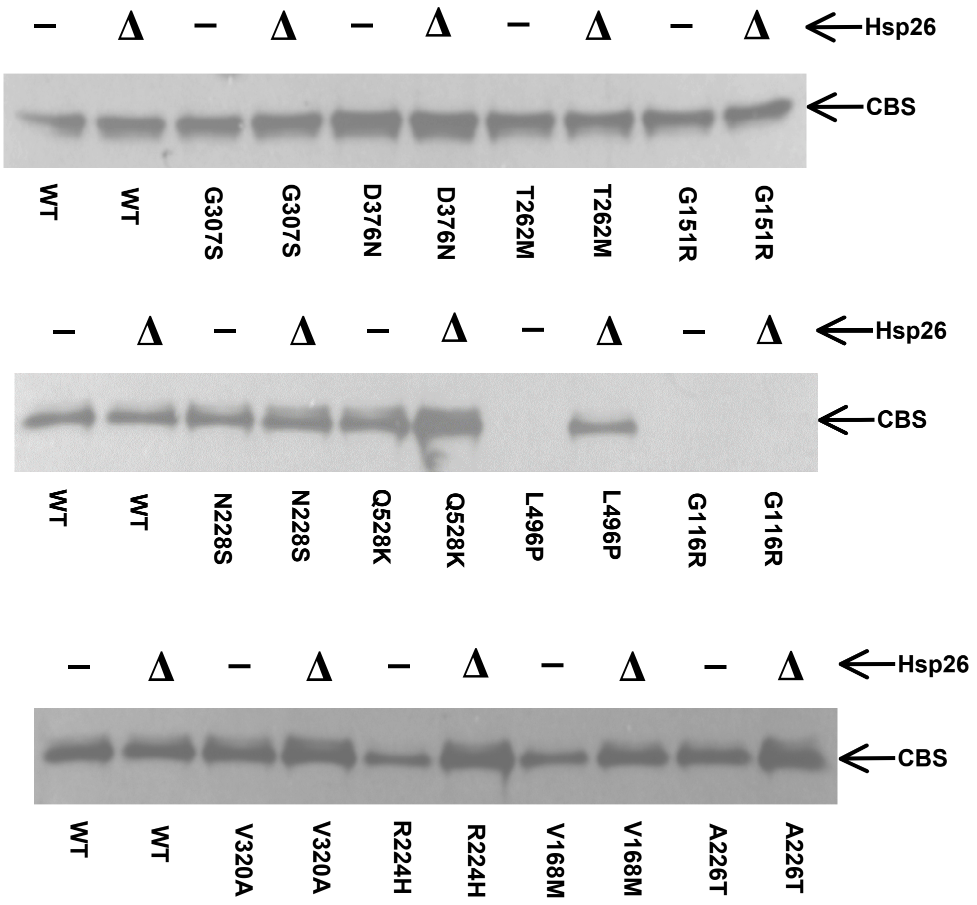

Supplement: Figure S2 — CBS protein levels in all mutants grown in the presence or absence of Hsp26. The indicated mutants were expressed in either a cys4Δ strain (Wy35) or a cys4Δhsp26Δ strain were grown in SC+CYS media, extracts were prepared, and Western analysis was performed using CBS antibody. (0.40 MB PDF) [file pgen.1000807.s002.pdf]
