# Supplementary material for: Activation of Mutant Enzyme Function In Vivo by Proteasome Inhibitors and Treatments that Induce Hsp70
Source: PLoS Genet. 2010 Jan 8;6(1):e1000807. doi: 10.1371/journal.pgen.1000807 (PMC2795852; doi:10.1371/journal.pgen.1000807)

Supp. Fig. 3. Interaction of D376N and G307S CBS with Hsp26. *(A)* The indicated mutants were expressed in either a *cys4Δ* strain (Wy35) or a *cys4Δhsp26Δ* (LS1) strain. Cells were grown in SC+CYS media, extracts were prepared, and Western analysis was performed using CBS, Hsp26, and α-tubulin anti-bodies. The CBS enzyme activity present in each extract is shown at the bottom (n=3; standard deviation shown). *(B)* Lysates from cells expressing the indicated human CBS allele were prepared and subject to immunoprecipitation using Hsp26 directed anti-body. Immunocomplexes were then analyzed by Western blot with CBS anti-bodies. Lane labeled control is extract without IP.

A.

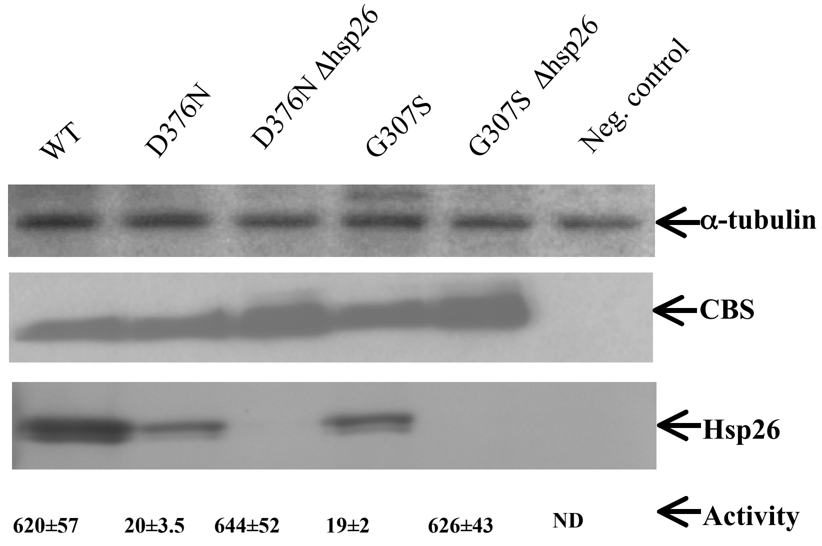

B.

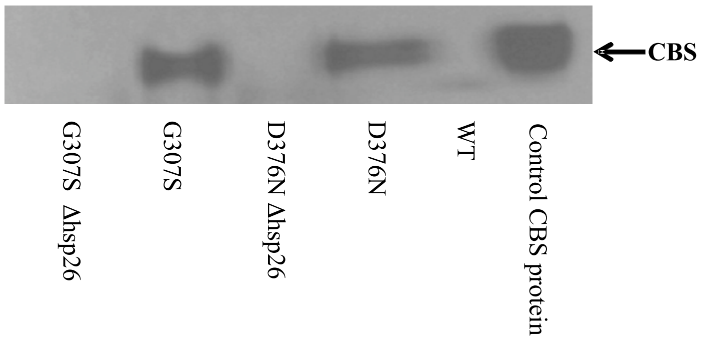

Supplement: Figure S3 — Interaction of D376N and G307S CBS with Hsp26. (A) The indicated mutants were expressed in either a cys4Δ strain (Wy35) or a cys4Δhsp26Δ (LS1) strain. Cells were grown in SC+CYS media, extracts were prepared, and Western analysis was performed using CBS, Hsp26, and α-tubulin anti-bodies. The CBS enzyme activity present in each extract is shown at the bottom (n = 3; standard deviation shown). (B) Lysates from cells expressing the indicated human CBS allele were prepared and subject to immunoprecipitation using Hsp26 directed anti-body. Immunocomplexes were then analyzed by Western blot with CBS anti-bodies. Lane labeled control is extract without IP. (0.19 MB PDF) [file pgen.1000807.s003.pdf]
