# Supplementary material for: Activation of Mutant Enzyme Function In Vivo by Proteasome Inhibitors and Treatments that Induce Hsp70
Source: PLoS Genet. 2010 Jan 8;6(1):e1000807. doi: 10.1371/journal.pgen.1000807 (PMC2795852; doi:10.1371/journal.pgen.1000807)

Supp. Fig. 4. CBS protein levels in all mutants grown in the presence or absence of bortezomib. The indicated mutants were expressed in a *cys4Δ* strain (Wy35) grown in SC+CYS media in the presence or absence of 50 μM bortezomib. After 24 hours, extracts were prepared and Western analysis was performed using CBS antibody.

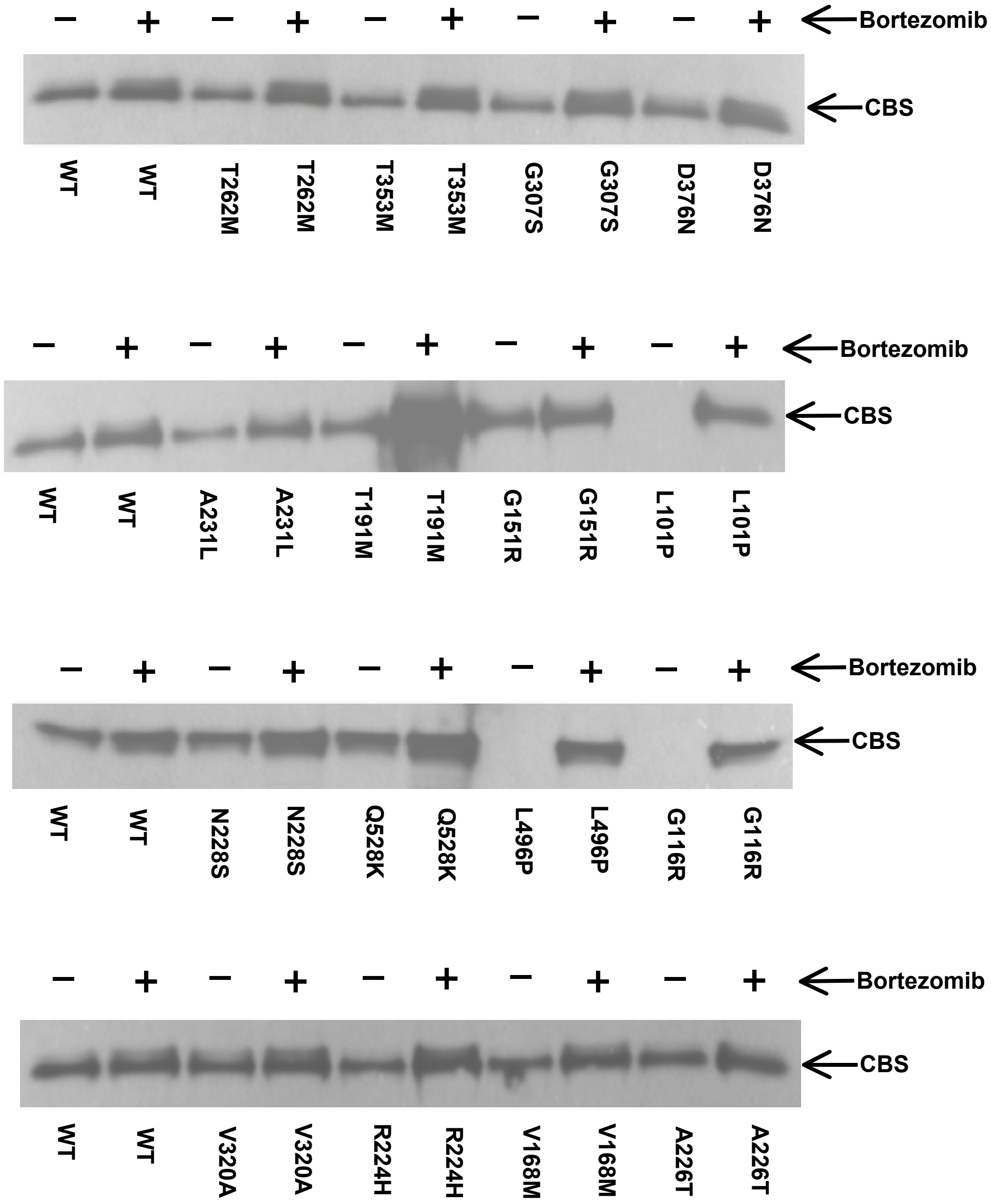

Supplement: Figure S4 — CBS protein levels in all mutants grown in the presence or absence of bortezomib. The indicated mutants were expressed in a cys4Δ strain (Wy35) grown in SC+CYS media in the presence or absence of 50 µM bortezomib. After 24 hours, extracts were prepared and Western analysis was performed using CBS antibody. (0.70 MB PDF) [file pgen.1000807.s004.pdf]
