# Supplementary material for: Activation of Mutant Enzyme Function In Vivo by Proteasome Inhibitors and Treatments that Induce Hsp70
Source: PLoS Genet. 2010 Jan 8;6(1):e1000807. doi: 10.1371/journal.pgen.1000807 (PMC2795852; doi:10.1371/journal.pgen.1000807)

Supp. Fig. 5. Effect of *ssa2Δ* and *hsp104Δ* on ethanol rescue of mutant p53. *(A) SSA2* or *ssa2Δ* yeast cells expressing the indicated p53 alleles were grown in either in the presence or absence of ethanol and assessed for growth in SC-ade-ura media after 24 hours by measuring $OD_{600.}$ *(B) HSP104* or *hsp104Δ* yeast cells expressing the indicated p53 alleles were grown in either in the presence or absence of ethanol and assessed for growth in SC-ade-ura media after 24 hours by measuring $OD_{600.}$

A.

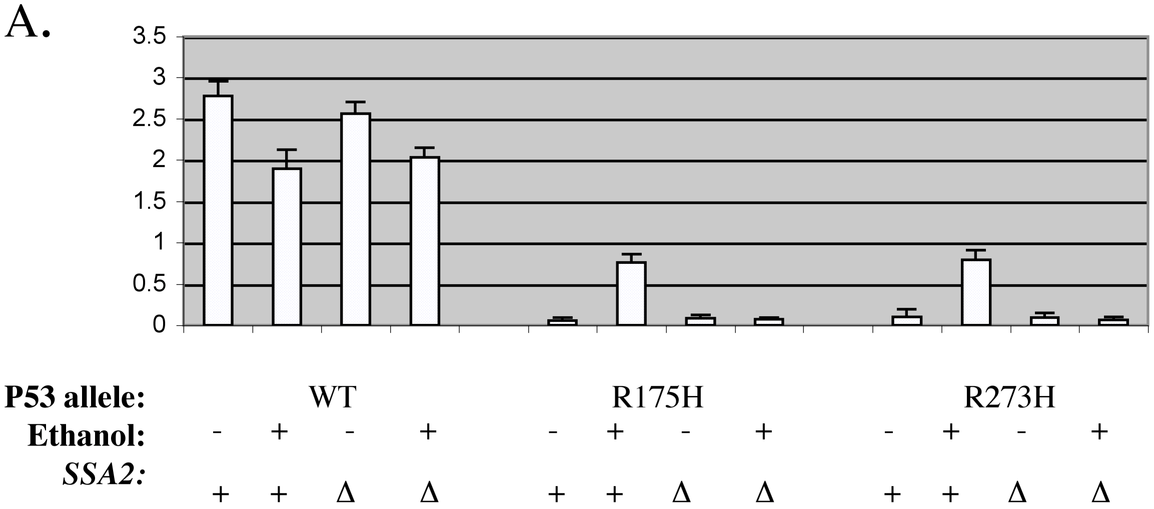

B.

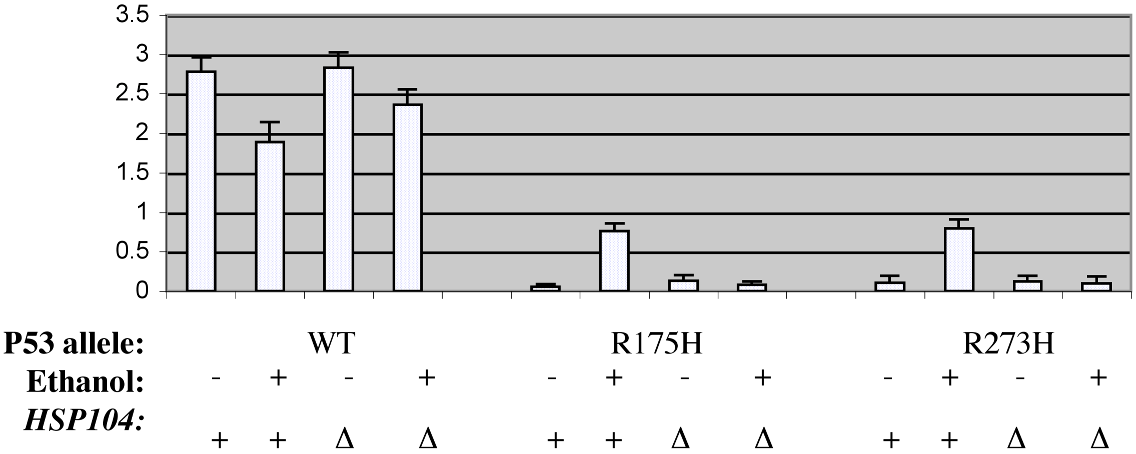

Supplement: Figure S5 — Effect of ssa2Δ and hsp104Δ on ethanol rescue of mutant p53. (A) SSA2 or ssa2Δ yeast cells expressing the indicated p53 alleles were grown in either in the presence or absence of ethanol and assessed for growth in SC-ade-ura media after 24 hours by measuring OD600. (B) HSP104 or hsp104Δ yeast cells expressing the indicated p53 alleles were grown in either in the presence or absence of ethanol and assessed for growth in SC-ade-ura media after 24 hours by measuring OD600. (0.16 MB PDF) [file pgen.1000807.s005.pdf]
