# Supplementary material for: Activation of Mutant Enzyme Function In Vivo by Proteasome Inhibitors and Treatments that Induce Hsp70
Source: PLoS Genet. 2010 Jan 8;6(1):e1000807. doi: 10.1371/journal.pgen.1000807 (PMC2795852; doi:10.1371/journal.pgen.1000807)

Supp. Fig. 6. Rescue of multiple mutant p53 by ethanol or bortezomib treatment. Stationary phase culture of yeast yIG397 expressing the indicated human p53 allele was diluted 1:1000 in SC–ade -ura media supplemented with either 4% ethanol or 50 μM bortezomib and grown at 30°C for 24 h. Growth was then determined by measuring OD at 600 nm.

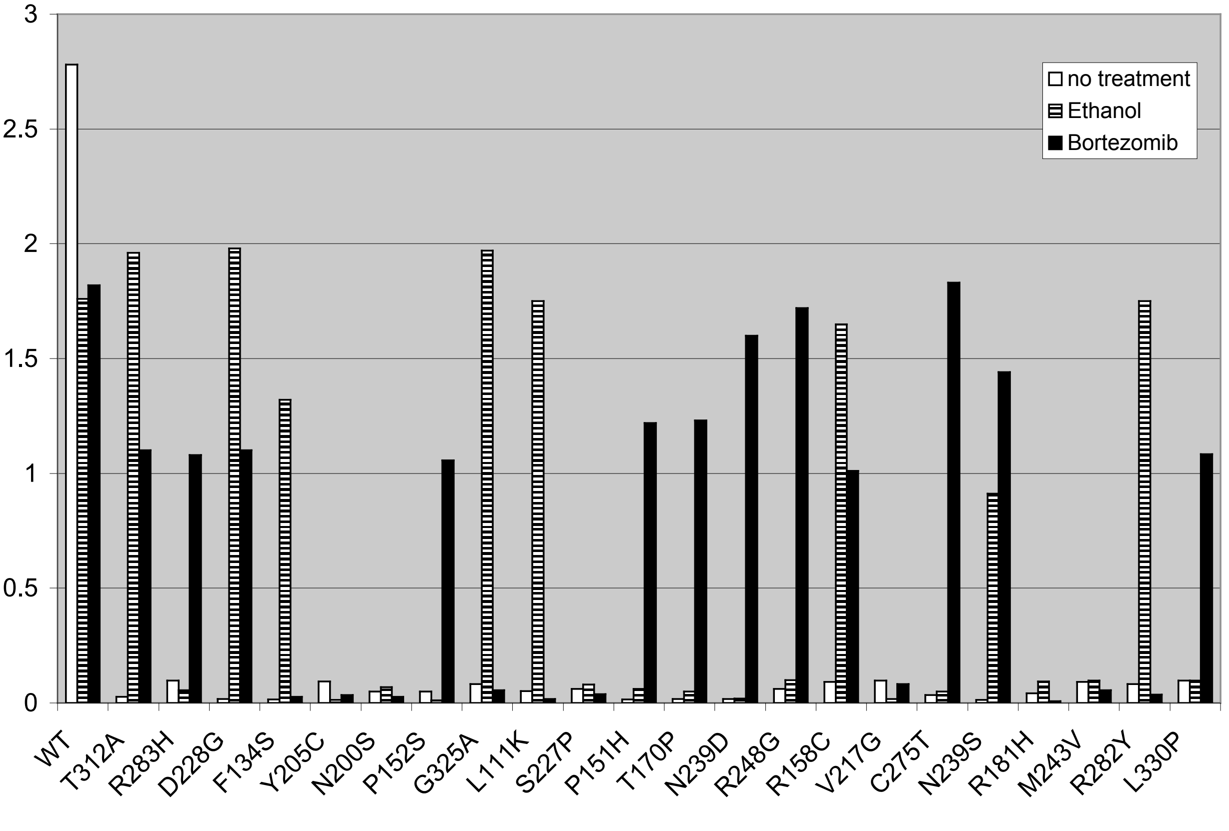

Supplement: Figure S6 — Rescue of multiple mutant p53 by ethanol or bortezomib treatment. Stationary phase culture of yeast yIG397 expressing the indicated human p53 allele was diluted 1∶1000 in SC–ade -ura media supplemented with either 4% ethanol or 50 µM bortezomib and grown at 30°C for 24 h. Growth was then determined by measuring OD at 600 nm. (0.07 MB PDF) [file pgen.1000807.s006.pdf]
