# Supplementary material for: Activation of Mutant Enzyme Function In Vivo by Proteasome Inhibitors and Treatments that Induce Hsp70
Source: PLoS Genet. 2010 Jan 8;6(1):e1000807. doi: 10.1371/journal.pgen.1000807 (PMC2795852; doi:10.1371/journal.pgen.1000807)

Supp. Fig. 7. CBS protein and activity in human cells treated with bortezomib. (A) Primary fibroblast cells expressing the indicated CBS allele were grown to 80% confluency at which time Bortezomib (18 μM) was added. After seven hours protein was harvested and Hsp70, CBS, and actin protein levels were measured by immunoblot. (B) Comparison of Hsp70 induction in bortezomib vs. MG132 cells. Gels were scanned using imaging software (Alpha Innotech 2.0) and the average induction is shown. Error bars show standard deviation. P <2.0×10^{-5} (t-test, two-sided).

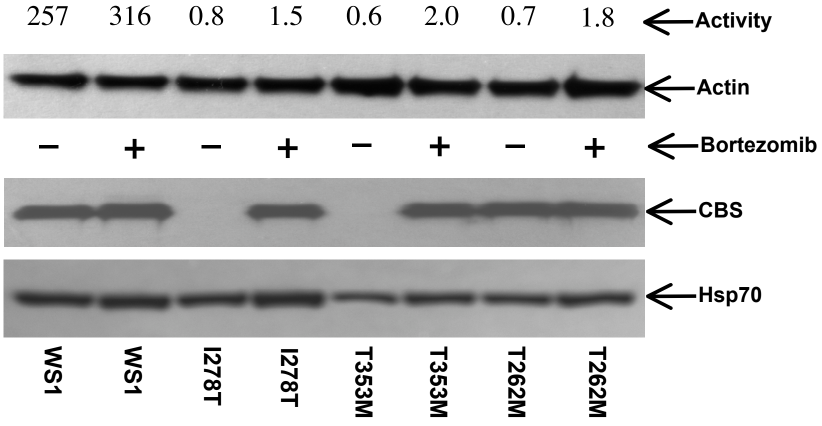

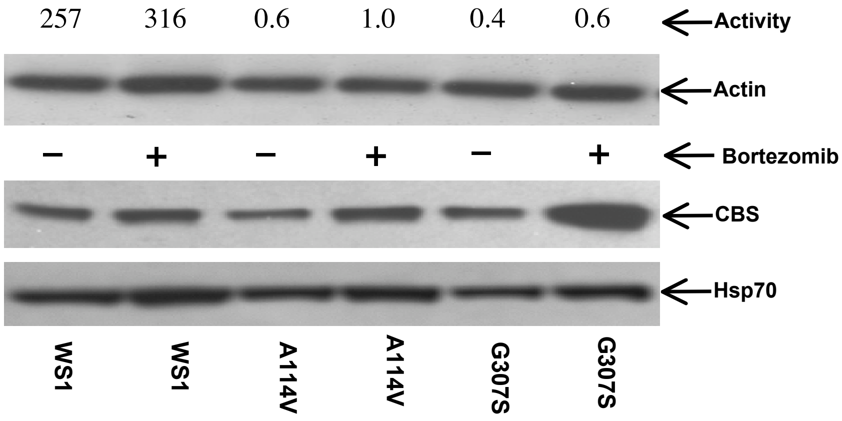

Supplement: Figure S7 — CBS protein and activity in human cells treated with bortezomib. (A) Primary fibroblast cells expressing the indicated CBS allele were grown to 80% confluency at which time Bortezomib (18 µM) was added. After seven hours protein was harvested and Hsp70, CBS, and actin protein levels were measured by immunoblot. (B) Comparison of Hsp70 induction in bortezomib vs. MG132 cells. Gels were scanned using imaging software (Alpha Innotech 2.0) and the average induction is shown. Error bars show standard deviation. P<2.0×10^−5^ (t-test, two-sided). (0.64 MB PDF) [file pgen.1000807.s007.pdf]
