# Supplementary material for: Activation of Mutant Enzyme Function In Vivo by Proteasome Inhibitors and Treatments that Induce Hsp70
Source: PLoS Genet. 2010 Jan 8;6(1):e1000807. doi: 10.1371/journal.pgen.1000807 (PMC2795852; doi:10.1371/journal.pgen.1000807)

Supp. Fig. 8. Serum total homocysteine and methionine levels in Tg-I278T Cbs-/- animals treated with bortezomib. Error bars show standard deviation *P<0.05 (t-test, 2-sided).

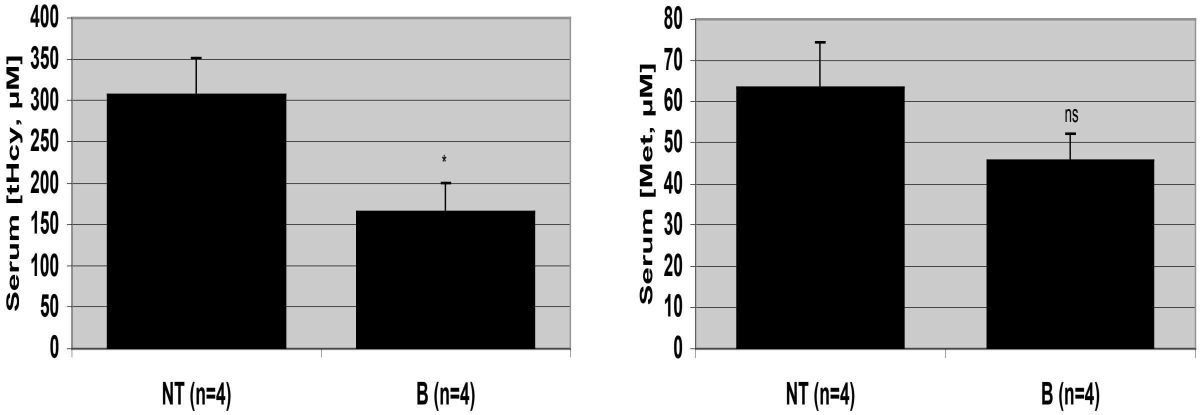

Supplement: Figure S8 — Serum total homocysteine and methionine levels in Tg-I278T Cbs−/− animals treated with bortezomib. Error bars show standard deviation *P<0.05 (t-test, 2-sided). (0.05 MB PDF) [file pgen.1000807.s008.pdf]
